# Biological Activity of New Cichoric Acid–Metal Complexes in Bacterial Strains, Yeast-Like Fungi, and Human Cell Cultures In Vitro

**DOI:** 10.3390/nu12010154

**Published:** 2020-01-06

**Authors:** Agata Jabłońska-Trypuć, Urszula Wydro, Elżbieta Wołejko, Grzegorz Świderski, Włodzimierz Lewandowski

**Affiliations:** Division of Chemistry, Biology and Biotechnology, Faculty of Civil Engineering and Environmental Sciences, Białystok University of Technology, Wiejska 45E Street, 15-351 Białystok, Poland; u.wydro@pb.edu.pl (U.W.); e.wolejko@pb.edu.pl (E.W.); g.swiderski@pb.edu.pl (G.Ś.); w.lewandowski@pb.edu.pl (W.L.)

**Keywords:** cichoric acid, metal complexes, cytotoxicity, cancer, bacterial strains, fungi, human cell culture

## Abstract

Cichoric acid (CA) belongs to the group of polyphenols, which occurs in a variety of plant species and it is characterized by anticancer, antibacterial, and antiviral properties. Selected polyphenols have the ability to combine with metal ions to form chelate complexes that reveal greater biological activity than free compounds. In order to study possible antimicrobial and anticancer effect of CA and its complexes with copper(II)/zinc(II)/nickel(II)/cobalt(II) we decided to conduct cytotoxicity tests to estimate the most effective concentrations of tested compounds. The results of the presented study demonstrated, for the first time, that the treatment with newly synthesized CA-metal complexes has anticancer and antimicrobial effects, which were examined in seven different cell lines: MCF-7, MDA-MB-231, and ZR-75-1 breast cancer cell lines, A375 melanoma cell line, DLD-1 cell line, LN-229 cell line, FN cell line; five bacterial strains: *Escherichia coli*, *Pseudomonas aeruginosa*, *Staphylococcus epidermidis*, *Proteus vulgaris, Lactobacillus rhamnosus*, yeast *Sacchcaromyces boulardii*, and pathogenic yeast-like fungi *Candida albicans*. The presented study indicates that CA-metal complexes could be considered as a potential supplementary tool in anticancer therapy, however, because of their possible toxic activity on fibroblasts, they should be used with caution. Some of the tested complexes have also preservative properties and positive influence on normal non-pathogenic microorganisms, which was demonstrated in selected microbial strains, therefore they may serve as food preservatives of natural origin with cytoprotective properties.

## 1. Introduction

Cichoric acid belongs to the group of polyphenols and according to its chemical formula it is a dicaffeyltartaric acid, a tartaric acid ester of two caffeic acids (a hydroxycinnamic acid). Because of its biological properties it is a very promising natural compound, which occurs in a variety of plant species such as *Cichorium intybus* L., *Ocimum basilicum* L., *Bidens tripartita* L., *Crepis capillaris* L. Wallr., *Lactuca sativa* L., *Taraxacum officinale* F.H. Wigg., *Cucurbita pepo* L., *Equisetum* hybrids, *Borago officinalis* L., *Posidonia oceanica* L. Delile, *Rabdosia rubescens*, and *Echinacea purpurea* [1]. According to the literature data cichoric acid plays an important role in plant defense against different diseases caused by viruses, bacteria, fungi, nematodes, and insects [2,3]. A variety of phenolics, including cichoric acid, are being investigated for their possible human health benefits. Cichoric acid (CA) is one of the polyphenol compounds with strong antioxidant capacities. It also exhibits free radical scavenger properties, antiviral and phagocyte promoting activity, and it protects selected structural proteins, such as collagen, against free radical damage [4]. CA has many biological activities including anti-inflammatory, antiatherosclerotic, and antidiabetic properties, which were confirmed in mouse models. Animal diabetes models indicated that CA significantly influenced insulin sensitivity, glucose tolerance, mitochondrial function, and hyperglycemia in obese mice [5,6,7]. 

Among the tested substances of natural origin, the ones that can be used in food as preservatives or pharmaceutical industry are the most popular. This is important in the context of the negative effects of drugs on human health and the increasing resistance of microorganisms to commercially available drugs and preservatives [1,8]. In addition, commonly used food preservatives such as nitrates, sodium benzoate, or monosodium glutamate may have potentially harmful effects on human health. Therefore, new alternatives that are easily accessible, safe for humans, and exhibiting antimicrobial activity are highly desirable in food industry and pharmacy. *Candida albicans*, *Escherichia coli*, *Proteus vulgaris*, *Pseudomonas aeruginosa*, and *Staphylococcus epidermidis* are examples of pathogen indicators that determine food quality and safety [8].

However, to date, a direct effect of metal complexes of CA on human cancer cells, normal healthy cells, and bacterial strains have not been investigated. Considering the stability of polyphenols, it is usually higher after binding them to a metal ion [5,9]. Also anticancer activity of metal complexes is widely used in therapy of different types of cancer; however, the utility of metal-polyphenols complexes as anticancer agents is yet to be fully realized, especially regarding some of the polyphenols such as cichoric acid [9].

According to the literature data, selected polyphenols have the ability to combine with metal ions to form chelate complexes that reveal greater biological activity than free compounds [10,11,12]. Out of all ions, copper seems very interesting and promising because of its biological importance and properties. It is essential in the photosynthesis process and respiration and it consists of an active center of many enzymes [13,14]. Therefore copper was one of the metals subjected to our research to determine biological activity of its complexes with CA. In complexes with fisetin–polyphenolic compound from the group of isoflavonoids, it revealed high antibacterial and antifungal activity [14]. On the other hand copper complexed with resveratrol acts as a factor, which promotes fragmentation of nuclear DNA in human cells [15]. Oleuropein, which is a non-flavonoid polyphenol, shows copper complexing properties and these copper–oleuropein complexes are probably involved in the toxic activity of analyzed polyphenolic compounds towards neuroblastoma cells, depending on their copper level [16]. The other important and biologically active metal ion which forms chelate complexes with polyphenolic compounds is zinc. Dias K et al. suggested that the combination of zinc with resveratrol enhances antioxidant activity of the polyphenolic compound [17]. The flavonoid derivative kaempferol in 1:1 coordination with Zn(II) increased phenol acidity of plant polyphenols. It may explain the very important and unique function of Zn(II) as a biologically active antioxidative compound and may help in designing new metal–polyphenol complex-based drugs derived from naturally occurring bioactive molecules [18].

Antioxidative, anti-inflammatory, and neuroprotective properties of polyphenols have been known for many years, but the discovery of their complexes with selected metals changed the course of medical chemistry and toxicology. In 2010 it was found out that complexes of biochanin with nickel(II) and copper(II) have antiviral, anticancer, and antioxidative properties [19]. Quercetin, which has in its structure hydroxyl groups capable of forming complexes with metal ions, chelates metals via 3′ or and 4′ phenolic group. Cobalt(II)–quercetin complexes have higher antioxidant activity than the free compound [20,21].

Our research group has shown in recent years that CA exhibits cytoprotective activity against Doxorubicin induced oxidative stress in human skin fibroblasts [22]. Furthermore we have shown that polyphenol–metal complexes exhibit better biological and antioxidative properties than free polyphenols [23]. Therefore, in order to study possible antimicrobial and anticancer effects of CA and its complexes with selected metals we decided to conduct a cytotoxicity tests to estimate the most effective concentrations of tested compounds.

## 2. Materials and Methods

### 2.1. Chemical Synthesis

#### 2.1.1. Sample Preparation

All reagents were from Sigma–Aldrich. Sodium salt of CA was prepared as follows: 9.96 mg of cichoric acid (MW = 474,371 g/mol) was weighed, i.e., 0.021 mmol, and dissolved in 0.42 mL NaOH solution at a concentration of 0.1 M. Deionized water (2 mL) was added to the mixture. The solution was stirred in a water bath at 50 °C to dissolve the acid. The molar ratio of ligand:metal was 1:2. The solution was allowed to evaporate slowly and the precipitate was air-dried at 30 °C.

The copper(II)/zinc(II)/nickel(II)/cobalt(II) complexes were prepared in the following way: 19.92 mg of cichoric acid was dissolved in 0.84 mL NaOH (0.1 M). Then 2 mL of deionized water and water solutions containing 0.084 mmol of copper(II), zinc(II), nickel(II), and cobalt(II) chlorides were added to the mixtures. The solutions were mixed with the use of a shaker for 2 hours at room temperature. In the obtained mixture, the molar ratio of sodium salt of cichoric acid to the transition metal cation was 1:2. After several days a precipitate occurred which was filtered from the solution on filter paper and washed with deionized water until the residual chlorides were washed out. The precipitate was air-dried at 30 °C. The yield of the synthesis processes was about 70–85%. Table 1 presents the results of elemental analysis. The sodium salt was dihydrated, while the complexes of copper, zinc, cobalt, and nickel were tetrahydrated.

#### 2.1.2. FTIR (Fourier Transform Infrared) Study

The FTIR spectra were recorded with an Alfa (Bruker) spectrometer within the range of 400–4000 cm^−1^. Samples in the solid state were measured in KBr matrix pellets. FT-Raman spectra of solid samples were recorded in the range of 400–4000 cm^−1^ with a MultiRam (Bruker) spectrometer.

#### 2.1.3. Calculations

To calculate the optimized structures quantum-mechanical methods were used: density functional (DFT) hybrid method B3LYP with non-local correlation provided by Lee–Young–Parr expression and HF (Hartree–Fock). All calculations were carried out with functional base 6-311++G(d,p). Calculations were performed using the Gaussian 09 (Frisch et al., 2009) package [24]. Experimental spectra were interpreted in terms of HF method calculations. Theoretical wavenumbers were scaled according to the formula: νscaled = 0.89·ν_calculated_ for HF/6-311++G(d,p) level. 

#### 2.1.4. Job’s Study

The composition of complexes in aqueous solutions was determined by the Job’s method. Chloride solutions of CoCl_2_, CuCl_2_, ZnCl_2_, and NiCl_2_ were prepared at concentrations of 0.01 M by dissolving the appropriate weight of metal chloride salts in deionized water. A solution of CA (0.1 M) was prepared by dissolving a sample of acid in 50 mL of Tris HCl buffer (pH = 7.2). To the 10 mL flasks, 5 mL of the CA solution was added, followed by the addition of a metal chloride solution in the range of 10 to 200 μL. Then the solutions were filled to 10 mL with Tris HCl buffer. The solutions were mixed and left for 1 hour. Then, the absorption spectra of the analyzed solutions in the UV range (190–400 nm) were recorded. The measurements were taken on a HACH 5000 DR spectrophotometer.

### 2.2. Toxicological Studies

#### 2.2.1. Reagents

Dulbecco’s modified Eagle’s medium (DMEM), containing glucose at 4.5 mg/mL (25 mM) with Glutamax and Leibovitz’s L-15 medium with Glutamax, penicillin, streptomycin, trypsin–EDTA, FBS (Fetal Bovine Serum) Gold, and PBS (Phosphate Buffer Saline) (without Ca and Mg) were provided by Gibco (San Diego, CA, USA). RPMI-1640 medium with high glucose and with L-glutamine was provided by ATCC. Cell Titer-Glo^TM^ 2.0 Assay was provided by Promega, Madison, WI, USA.

#### 2.2.2. Microbial Strains

*Escherichia coli* (ATCC 25922), *Pseudomonas aeruginosa* (ATCC 27853), *Staphylococcus epidermidis* (ATCC 12228), *Candida albicans* (ATCC 10231), *Saccharomyces boulardii*, and *Lactobacillus rhamnosus* (ATCC 53103) were obtained from the American Type Culture Collection (Manassas, VA, USA). *Proteus vulgaris* (PCM 2269) strain was purchased from Polish Collection of Microorganisms (PCM, Wroclaw, Poland). Strains of bacteria and fungi were selected for antimicrobial tests. *E. coli*, *P. aeruginosa*, *P. vulgaris* (Gram negative bacteria), *S. epidermidis*, *L. rhamnosus* (Gram-positive bacteria), and *C. albicans* and *S. boulardii* (fungi) were grown overnight in Mueller Hinton II Broth at 37 °C (*E. coli*, *P. vulgaris and S. epidermidis*, *L. rhamnosus*, *S. boulardii*) and 27 °C (*P. aeruginosa* and *C. albicans*). Next day, the overnight cultures were diluted in fresh MH II Broth to obtain 10^8^ CFU/mL (CFU—colony forming units). For the antimicrobial activity, the inoculum of the tested bacteria reached the final concentration value of 10^6^ CFU/mL, while the inoculum of *C. albicans* and *S. boulardii* were about 10^4^ CFU/mL. 

#### 2.2.3. Cell Culture

The effect of CA and its metal complexes were examined in MCF-7, MDA-MB-231, and ZR-75-1 breast cancer cell lines, A375 melanoma cell line, DLD-1 cell line, LN-229 cell line, and FN cell line, which were obtained from American Type Culture Collection (ATCC). MCF-7 cells, A-375 melanoma cell line, LN-229 glioblastoma cell line, and FN fibroblasts cell line were maintained in DMEM supplemented with 10% FBS, penicillin (100 U/mL), and streptomycin (100 μg/mL) at 37 °C in a humidified atmosphere of 5% CO_2_ in air. ZR-75-1 cells and DLD-1 colorectal adenocarcinoma cell line were maintained in RPMI-1640 supplemented with 10% FBS, penicillin (100 U/mL), and streptomycin (100 μg/mL) at 37 °C in a humidified atmosphere of 5% CO_2_ in air. MDA-MB-231 cells were maintained in Leibovitz’s L-15 medium supplemented with 10% FBS, penicillin (100 U/mL), and streptomycin (100 μg/mL) at 37 °C in a humidified atmosphere. 

MCF-7 cells, MDA-MB-231 cells, ZR-75-1 cells, A-375 cells line, DLD-1 cells, LN-229 cells, and FN cells (2 × 10^4^ cells/ml) in 200 µL of culture medium were incubated without and with the test compounds in tissue culture treated white 96-well plates for the Cell Titer Glo™ 2.0 Assay. The cytotoxicity was estimated for CA and its complexes at concentration of 50 µM, 100 µM, 200 µM, 300 µM, 400 µM, and 500 µM. 

#### 2.2.4. CA and Its Metal Complexes Antimicrobial Activity

Initially, two-fold microdilutions of analyzed compounds in a liquid growth media (MH II Broth) in 96 well-plates were prepared. Next, the indicator fungi (concentration of 10^4^ CFU/mL) and bacteria strain (concentration of 10^6^ CFU/mL) suspensions were added into each well of a 96 well-plate, and then incubated for 24 h at 37 °C (*E. coli*, *P. vulgaris*, *S. epidermidis*, *L. rhamnosus*, and *S. boulardii*) and 27 °C (*P. aeruginosa and C. albicans*). The final concentrations of CA and its complexes in each well were: 800 µM, 400 µM, 200 µM, 100 µM, and 50 µM.

Cell viability of tested microorganisms treated with CA and its metal complexes were estimated using the BacTiter-Glo™ (Promega, Madison, WI, USA) according to the manufacturer’s instruction. In brief, the assay uses a thermostable luciferase to enable reaction conditions that produce a stable “glow-type” luminescent signal while simultaneously inhibiting endogenous enzymes released during cell lysis. The homogenous assay procedure involves addition of a single reagent directly to cells cultured in serum-supplemented medium. Luminescence was measured with a GloMax^®^-Multi Microplate Multimode Reader. The cytotoxicity of CA and its complexes with metals was expressed a relative cell viability (%) in relation to un-treated control. The study was performed in triplicate in order to ensure that consistent results were obtained.

#### 2.2.5. Estimation of CA and Its Metal Complexes Cytotoxicity

To measure CA and metal complexes cytotoxicity CellTiter-Glo™ 2.0 Assay (Promega) was used. The measurement was conducted according to manufacturer’s protocol. Luminescence was measured with a GloMax^®^-Multi Microplate Multimode Reader. The study was performed in triplicate taken to ensure consistent results were obtained. 

#### 2.2.6. Statistical Analysis

All data are given as mean values ±SD (standard deviation). Differences between treatments and untreated control human cells were analyzed by one-way ANOVA, followed by Dunnett’s procedure for multiple comparisons. Significant effects are represented by *p* ≤ 0.05 (*), *p* ≤ 0.01 (**), *p* ≤ 0.001 (***). To compare the means for treatments and tested cell lines two-way analysis of variance (ANOVA) followed by the Tukey test were applied. Significance was considered when p ≤ 0.05. Cluster analysis was used to the group the examined elements (individual microorganisms treated with metal, CA and its complexes with metal) into similar categories. Two-way joining method of clustering was applied. 

## 3. Results

### 3.1. Chemical Synthesis Results

#### Composition and Structure of Examined Complexes

Elemental analysis showed that the metals Zn(II), Cu(II), Ni(II), and Co(II) are complexed with cichoric acid in a 2:1 molar ratio (metal:ligand) (Table 1). All complexes were hydrated and contained 4 water molecules. The yield of the synthesis oscillated around 70%–80% (based on the synthesis of complexes three times).

**Figure 1:**
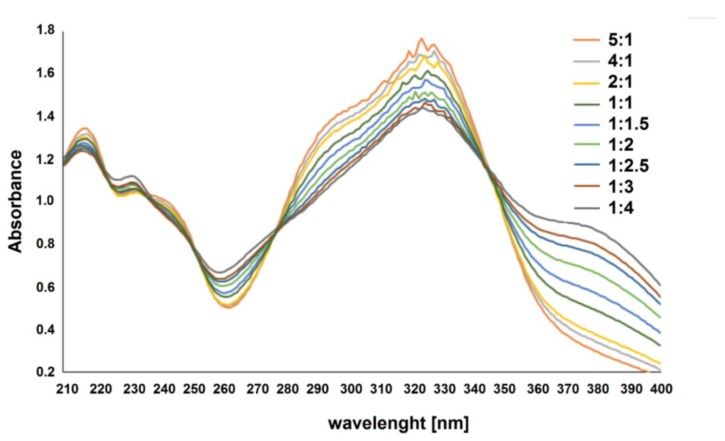
Absorption spectra of cichoric acid in the presence of various amounts of copper in Tris–HCl buffer pH 7.2 (different types of lines are labeled with a molar ratio of cichoric acid:metal 5:1–1:5).

**Figure 2:**
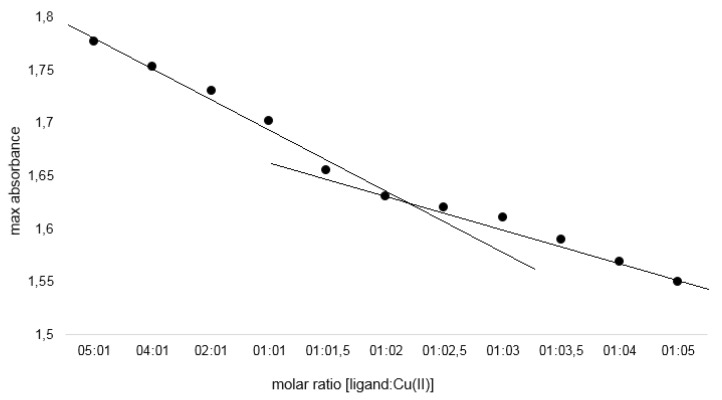
Absorbance as a function of [cichoric acid]: [copper] molar ratio at 320 nm.

The composition of the complexes in aqueous solutions was determined using the Job’s method for pH = 7.2. The analysis showed that in aqueous solutions cichoric acid complexes with metals in a molar ratio of 1:2. Figure 1 presents UV spectra for aqueous solutions of cichoric acid with copper (II) ions (mixed in molar ratios from 5:1 to 1:5) measured in a Tris–HCl buffer solution with pH = 7.2. Figure 2 shows the dependence of the maximum absorbance of a copper complex with cichoric acid depending on the composition of the complex. Similar results were noted for the remaining studied metal complexes with cichoric acid.

The type of metal ligand coordination was determined based on spectroscopic data analysis (FTIR). The spectra of cichoric acid and metal complexes were recorded in the KBr matrix, and theoretically calculated for the optimized structure of the acid, sodium salt, and copper complex.

**Figure 3:**
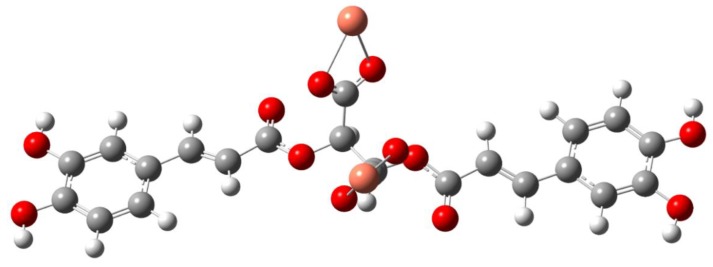
The propoedl structures of copper and cobalt cichoriate structures.

**Figure 4:**
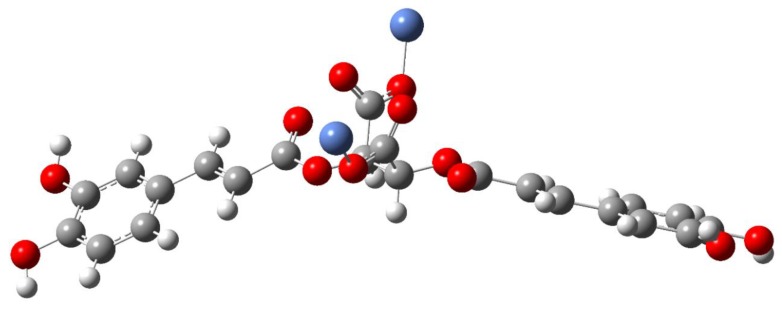
The proposed structures of nickel and zinc cichoriate structures.

In the spectrum of the acid the bands at 1746 and 1716 cm^−1^ were interpreted as vibrations of the carboxyl group of the tartaric acid fragment present in the cichoric acid molecule. After binding the metal ion to the ligand, these bands disappeared, which indicates that the metal is bound to the cichoric acid through the carboxylate group of the tartaric acid. In the spectra of the sodium salt and complexes, characteristic wide bands originating from vibrations of the carboxylate anion appeared. The bands derived from the stretching vibrations of the symmetric carboxylate anion ν_sym_COO^−^ were identified: at the wavenumbers: 1385 cm^−1^ IR and 1381 cm^−1^ Raman in sodium salt, 1384 cm^−1^ IR in the copper complex, 1385 cm^−1^ IR in the nickel complex, 1384 cm^−1^ IR in the zinc complex, and 1385 cm^−1^ IR in the complex cobalt, and stretching asymmetric ν_as_COO: at the wavenumbers: 1626 cm^−1^ IR in sodium salt, 1626 cm^−1^ IR in the copper complex, 1630 cm^−1^ IR in the nickel complex, 1629 cm^−1^ IR in the zinc complex, and 1605 cm^−1^ IR in the cobalt complex (Table 2). In the spectra of complexes intense bands assigned to the symmetric bending in-plane (β_s_COO^−^) occurred: at the wavenumbers: at 853 cm^−1^ IR and 860 cm^−1^ Raman in sodium salt, 868 cm^−1^ in the copper complex, 851 cm^−1^ in the nickel complex, 853 cm^−1^ in the zinc complex, and 851 cm^−1^ in the cobalt complex, and asymmetric bending β_as_COO^−^: at 521 cm^−1^ IR in sodium salt, 521 cm^−1^ in the copper complex, 520 cm^−1^ in the nickel complex, 520 cm^−1^ in the zinc complex, and 521 cm^−1^ in the cobalt complex (Table 2). On the basis of the position of the ν_as_COO^−^ and ν_s_COO^−^ bands in the IR spectra of the studied complexes compared to the position of these bands in the sodium salt, it was found that the bidentate chelation coordination mode is present in the copper and cobalt complexes (Figure 3) and monodentate for nickel and zinc complex (Figure 4) (ΔνCOO^−^ of the complexes < ΔνCOO^−^ of the sodium salt; ΔνCOO^−^ means the difference between the wavenumber of the bands assigned to the asymmetric and symmetric vibration of the carboxylate anion).

### 3.2. Toxicological Studies Results

#### 3.2.1. Antibacterial and Antifungal Activity of CA and Its Metal Complexes

**Figure 5:**
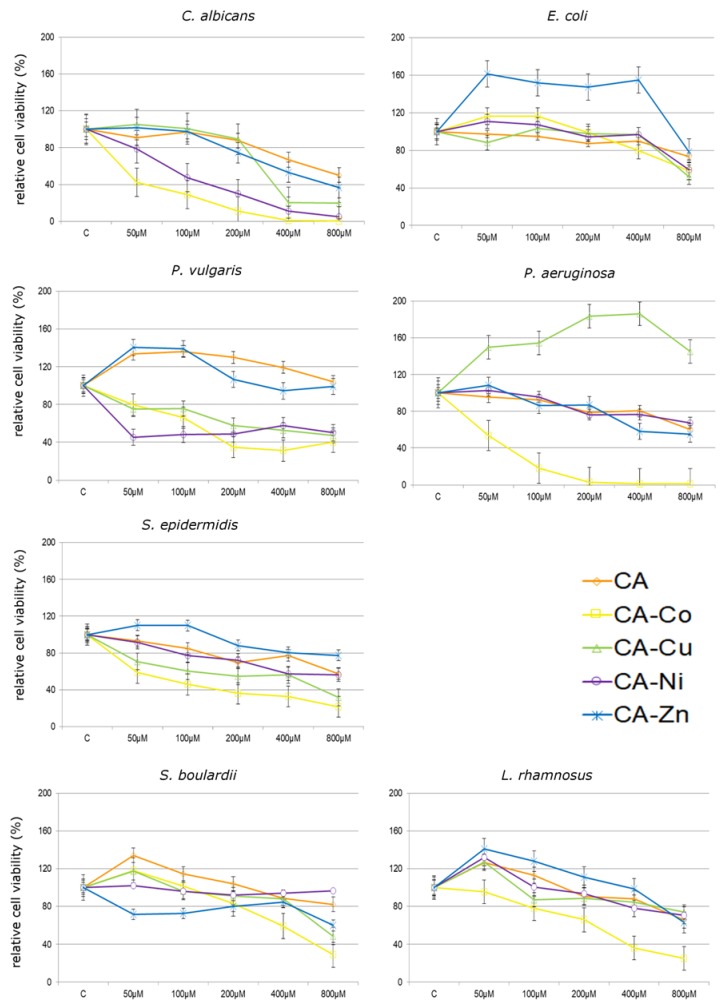
Cytotoxicity of cichoric acid (CA) and its complexes with Co (CA–Co), Cu (CA–Cu), Ni (CA–Ni) and Zn (CA–Zn) on fungi (*C. albicans* and *S. boulardii*) and bacteria strains (*E. coli*, *P. vulgaris*, *P. aeruginosa*, *S. epidermidis*, *L. rhamnosus*) expressed as relative cell viability (%) as compared to non-treated control (C). Each value on the graph is the mean of three independent experiments and error bars show the standard deviation (SD).

**Figure 6:**
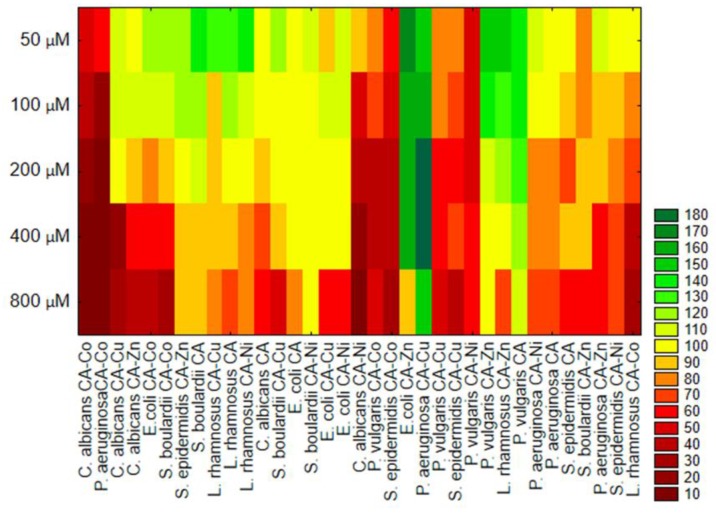
Cluster analysis for relative fungi and bacteria cells viability (%) after treatment of CA and its complexes with metals.

Figure 5 and Figure 6 show the influence of CA and its complexes with selected metals on all tested microbial and fungal strains. In all analyzed concentrations, *P. aeruginosa* incubation with CA–Co complex leads to statistically significant decreases in cell viability. The lowest tested concentration of CA–Co complex of 50 µM caused a decrease of approximately 50% after 24 h treatment. At higher concentrations of CA–Co complex, i.e., from 200 µM to 800 µM, decreases in cell viability by approximately 97% as compared to the control untreated cells were observed. Similar effects were noticed in the case of CA–Co treatments in *S. epidermidis* and *P. vulgaris*, in which all applied concentrations caused decreases in relative cell viability in *S. epidermidis* from 40% at 50 µM to 80% at 800 µM and *P. vulgaris* from 20% at 50 µM to 70% at 400 µM. In turn, using the lowest concentration of CA–Co complex caused an increase in relative cell viability of *E. coli* of approx. 20%. 

At all investigated treatments, the CA–Cu and CA–Ni complexes resulted in a decrease in cell viability in both *S. epidermidis* and *P. vulgaris*. The CA–Cu complex in all analyzed concentrations caused an increase in *P. aeruginosa* viability by approximately 50% as compared to the control untreated cells. The treatment of *E coli* with CA–Cu and CA–Ni did not cause any significant decreases in relative cell viability at lower tested concentrations. The decrease was observed only at 800 µM in relative cell viability by about 40% and 50% respectively. After introducing CA–Ni and CA–Zn complexes on *P. aeruginosa* we observed a decrease in cell viability with simultaneous increase in concentrations used. In the case of *S. epidermidis*, at lower tested concentrations of CA–Zn complex (50 and 100 µM) there was an increase in the viability by about 7%, while at other concentrations, i.e., from 200 to 800 µM a decrease by approximately 15% at 200 µM, 20% at 400 µM, and 25% at 800 µM was observed. The highest concentration of CA–Zn complex caused over 20% decline in *E. coli* cell viability, whereas lower concentrations stimulated cell viability by nearly 60% after 24 h incubation. 

The application of CA and its complexes with selected metals on *C. albicans* caused a decrease in relative cell viability after 24 h. The highest (800 µM) concentration of CA complexed with selected metals caused a decrease in relative cell viability by approximately 60% for CA–Zn, 80% for CA–Cu, and 95% for CA–Co and CA–Ni as compared to the control untreated cells. In CA–Cu and CA–Zn, the lowest analyzed concentration did not cause any significant changes in relative cell viability, since it achieved the value of approximately 3% (Figure 5). 

The application of CA on nonpathogenic microorganisms such as *L. rhamnosus* and *S. bouldardii* caused an increase in relative cell viability in *L. rhamnosus* by approximately 26% at 50 µM and 12% at 100 µM and in *S. bouldardii* by approximately 34% at 50 µM, 14% at 100 µM, and 4% at 200 µM. After introducing CA–Zn complexes on *L. rhamnosus* an increase in relative cell viability from 40% at 50 µM to 10% at 200 µM was observed, while in *S. bouldardii* a decrease in relative cell viability from 29% at 50 µM to 40% at 800 µM was noticed. *L. rhamnosus* incubation with CA–Co complexes lead to decreases in cell viability after 24 h treatment as compared to the control untreated cells. In turn, using the 50 µM CA–Co and CA–Cu complex caused an increase in relative cell viability of *S. bouldardii* by approximately 18%. Similar effects were noticed in the case of CA–Cu and CA–Ni complexes, i.e., by approximately 30% in *L. rhamnosus.*

Summarizing, cytotoxic effect of tested compounds on selected microorganisms decreases in the following sequence:

*C. albicans*: Ca–Co > Ca–Ni > Ca–Cu > Ca–Zn > CA

*E. coli*: CA–Co > CA–Cu > CA > CA–Ni > CA–Zn

*P. vulgaris*: CA–Ni > CA–Co > CA–Cu > CA–Zn > CA

*P. aeruginosa*: CA–Co > CA–Zn > CA >CA–Ni > CA–Cu

*S. epidermidis*: CA–Co > CA–Cu > CA–Ni > CA > CA–Zn.

Based on resulted of cluster analysis (Figure 6), we can distinguish the groups that contain microorganisms with similar sensitivity/resistance to individuals treatments. For example, the similar highest inhibitory effect was observed for *C. albicans*, *P. aeruginosa*, and *S. epidermidis* treatment with the CA–Co complex. On the other hand, the similar stimulatory effects for *E. coli* treated with CA–Zn, *P. aeruginosa* with CA–Cu, and *P. vulgaris* with CA and CA–Zn were noted. 

#### 3.2.2. Cytotoxicity of Analyzed Compounds in Human Cancerous and Non-Cancerous Cells

**Figure 7:**
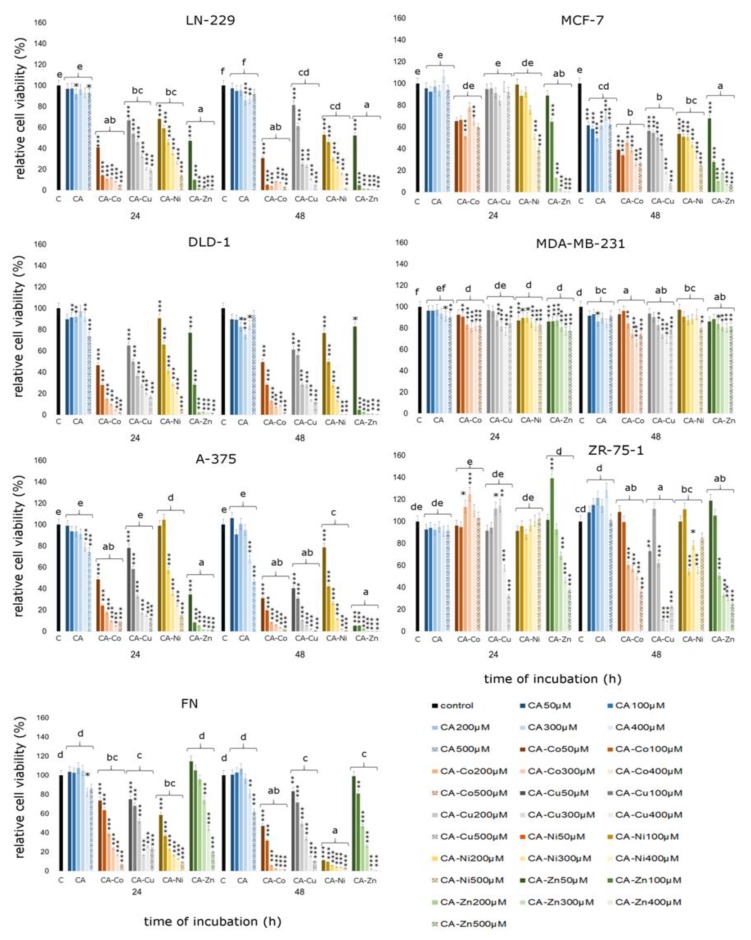
Cell viability results for MCF-7, MDA-MB-231, ZR-75-1, A375, DLD-1, LN-229, and FN cell lines exposed to different concentrations of CA and CA complexed with metals (Cu, Zn, Co, Ni) for 24 h and 48 h calculated as a percentage of control, untreated cells. Each value on the graph is the mean of three independent experiments and error bars show the standard deviation (SD). * *p* < 0.05, ** *p* < 0.01, and *** *p* < 0.001 represent significant effects between treatments and control followed by a Dunnett’s test.

As shown in Figure 7 both CA and its complexes with selected metals exert cytotoxic effect in all tested cell lines. In the LN-229 glioblastoma cell line incubation with especially CA–Co complexes in all analyzed concentrations leads to statistically significant decreases in cell viability. Even the lowest tested concentration of CA–Co complexes—50 µM—caused a decrease by about 60% after 24 h treatment and by about 70% after 48 h treatment. A similar effect was observed in the case of CA–Zn treatment in both incubation times. The lowest analyzed concentration caused a decrease in relative cell viability by about 50% after 24 h and 48 h treatment. Higher concentrations of CA–Zn complex caused more than 90% decline in LN-229 cells viability. CA treatment did not cause any significant decreases in relative cell viability in LN-229 cell line. DLD-1 colorectal adenocarcinoma cells treated with CA and CA–metal complexes also exhibited statistically significant decreases in relative cell viability. An effect comparable with the LN-229 cell line was observed. CA complexed with Co already in the lowest concentration caused almost 50% decline in DLD-1 cell viability after 24 h incubation. Ca complexed with Zn in both analyzed incubation times caused statistically significant decreases in cell viability in all tested concentrations. The most inhibitory effect on cell viability was noticed for CA–Co and CA–Zn complexes causing decreases higher than 90%. In the case of CA–Zn complex all of the analyzed concentrations, except 50 µM, caused decline in cell viability. The influence of CA on the DLD-1 cell line was more significant that on the LN-229 cell line; however we did observe significant effects only in higher concentrations such as 300 µM. In the A-375 melanoma cell line CA–metal complexes also caused significant decreases in cell viability. Co, Zn, and Cu complexes were characterized by the greater inhibitory activity towards melanoma cells. After 48 h treatment, the CA–Zn complex in the lowest concentration of 50 µM caused a decrease by about 95% in relative cell viability. Cytotoxicity assay revealed also that statistically significant changes in cell number were observed in analyzed breast cancer cell lines, especially in the MCF-7 cell line after 48 h treatment. CA–metal complexes were more cytotoxic to breast cancer cells than free cichoric acid. An especially active compound was CA–Zn complex in 200 µM, 400 µM, and 500 µM concentration. In the MDA-MB-231 cell line we did observe only slight significant changes in cell viability under the influence of tested compounds. Only the CA–Co complex in 400 µM concentration after 48 h treatment caused significant changes in cell viability. In the ZR-75-1 cell line CA–Cu and CA–Zn were especially active in 400 µM and 500 µM concentrations. In fibroblasts, which represent normal, healthy cells, significant declines in cell viability were observed. Only free CA exhibited stimulatory activity against fibroblasts. It caused insignificant increases in relative cell viability in the concentration range 50 µM to 300 µM. Higher concentrations such as 400 µM and 500 µM inhibited cell proliferation by about 20% as compared to control untreated cells.

**Figure 8:**
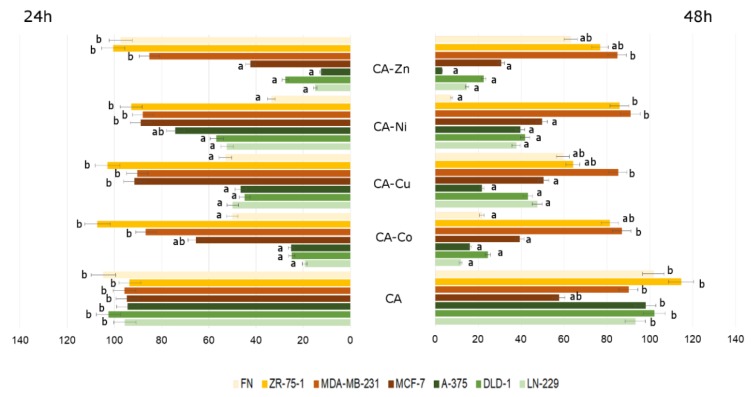
The viability of LN-229, DLD-1, A-375, FN, MCF-7, MDA-MB-231, and ZR-75-1 cell lines treated with different concentrations of CA and CA–metal (Cu, Zn, Ni, Co) complexes for 24 and 48 h. The results represent means for pooled triplicate values from three independent experiments. Significant alterations are expressed relative to control untreated cells (marked with asterisks). Statistical significance was considered if * *p* < 0.05.

Summarizing, the influence of CA and CA-metal complexes on cancerous and non-cancerous cells is depicted in Figure 8. Presented results are shown as a percentage of live cells compared to control, untreated cells set at 100 percent, with no regard both to the concentration of CA or to the concentration of CA-metal complexes. The differences in relative cell viability are especially easily visible in CA–metal complexes. In case of CA treatment, significant changes and differences between analyzed cell lines were observed after 48 h incubation. From the presented graph it can be deducted that every analyzed CA–metal complex, without considering concentration, caused decrease in cell viability in three cancer cell lines: A-375, DLD-1, and LN-229. Interestingly, breast cancer cell lines, especially MDA-MB-231 and MCF-7, were more resistant to CA–metal complexes as compared to other cancer cell lines and to ZR-75-1 breast cancer cell line. Unfortunately, CA–metal complexes, especially CA–Ni and CA–Co after 48 h incubation, were cytotoxic to fibroblasts, which represent normal healthy cell lines. Free CA did not exhibit toxicity against fibroblasts and after 48 h treatment it was toxic to the MCF-7 cell line. In every tested concentration, except for free CA, after 48 h treatment the level of viable cells did not exceed 100% control untreated cells.

Summarizing, cytotoxic effect of tested compounds on selected human cancerous and non-cancerous cell lines decreases in the following sequence:

LN-229: CA–Zn > CA–Co > CA–Ni > CA–Cu > CA

DLD–1: CA–Zn > CA–Cu > CA–Co > CA–Ni > CA

A–375: CA–Zn > CA–Co > CA–Cu > CA–Ni > CA

MCF–7: CA–Zn > CA–C > CA–Ni > CA–Cu > CA

MDA–MB–231: CA–Zn > CA–Co > CA–Cu > CA–Ni > CA

ZR–75–1: CA–Cu > CA–Zn > CA–Ni > CA–Co > CA

FN: CA–Ni > CA–Co > CA–Cu > CA–Zn > CA

## 4. Discussion

Cichoric acid, due to its numerous beneficial biological properties, is a valuable product of natural origin. Literature data indicate its immunostimulatory and antiviral activity, and the capacity of stimulating the effect of phagocytosis in vitro and in vivo. In addition, CA inhibits hyaluronidase activity, which is a key enzyme in the course of bacterial infection [25]. In the present study we examined the effect of CA and its complexes with metals against foodborne pathogenic microorganisms such as *E. coli*, *P. vulgaris*, *P. aeruginosa*, *S. epidermidis*, and *C. albicans* as well as nonpathogenic microorganisms like *L. rhamnosus* and *S. boulardii.* In literature, there are limited numbers of studies about the influence of CA on bacteria or fungi. However, several data indicate that polyphenolic acids have antibacterial and antifungal properties against foodborne pathogens [26,27,28]. Furthermore, it has been reported that chemical complexes with selected heavy metals such as Co, Cu, Zn, and Ni may improve antibacterial and antifungal potential [29]. 

According to obtained results, antimicrobial effect varies depending on studied microorganism as well as on applied agent. Generally, CA complexed with Co was the strongest antibacterial and antifungal compound, whereas the least efficient in reducing tested bacteria and fungi viability were CA and CA–Zn complexes. Inhibitory or stimulatory effects of studied compounds on individual bacterial strains and fungi may be explained by the different structure of cell membranes, as reported by many authors [30,31]. Gram-negative bacteria are generally most resistant to antibacterial compounds due to the presence of the outer cell membrane. In Gram-negative bacteria the molecules are transported through the outer lipopolysaccharide membrane rich in porins, which significantly hinder the penetration of antibacterial agents. Our data indicate that free CA exhibited a similar impact against *S. epidermidis* (Gram-positive) and *P. aeruginosa* (Gram-negative). These results are not consistent with previous studies reporting that polyphenolic acids have a higher antibacterial potential against Gram-positive than Gram-negative bacteria [28]. However, research conducted by Cueva et al. showed that selected phenolic compounds (e.g., gallic acid, caffeic acid) may inhibit the growth of both Gram-positive (*S. aureus*) and Gram-negative bacteria (*P. aeruginosa*), which is in agreement with our results regarding to CA [32]. Kavak and Kacec (2019) also drew similar conclusions in study about arbutin (polyphenols extracted from *Pyrus elaeagrifolia*), which was an effective antibacterial agent against *Bacillus cereus* (Gram-positive), *Staphylococcus aureus* (Gram-positive), and *Escherichia coli* (Gram-negative) [30]. According to Mokhtar et al. antimicrobial properties of some polyphenolic acids may be associated with irreversible changes in the structure and properties of the bacteria cell membrane [8]. These compounds may cause changes in hydrophobicity and the formation of local ruptures or pores resulting in leakage of cell components. Furthermore, polyphenols can destroy the bacteria membrane proteins and disrupt bacterial metabolic processes [33]. In addition, Lou et al. (2015) showed that phenolic acids like p-coumaric acid may increase membrane permeability as well as may bind to genomic DNA and disrupt major processes such as replication, transcription, and translation [34].

In the present study we tested antifungal properties of free CA. It was found that CA inhibited fungi cell growth by approximately 20% as compared to control. It has been reported the transfer of molecules is associated with the ultrastructure of the chitin wall, membrane ergosterols, and genetic material. Therefore, a complex structure of fungal cell walls could hinder the transport of antifungal agents [31].

In our research free cichoric acid was not effective in reducing the tested microorganisms’ growth. The maximum inhibitory effect of CA (20%) was observed for *S. epidermidis*, *C. albicans*, and *P. aeruginosa*. As reported by Prasad et al. and Świsłocka et al. (poli)phenolic compounds are characterized by low antimicrobial activity as compared to their combinations with other molecules [23,35]. In our research we proposed determining the antimicrobial properties of one from polyphenol acids, cichoric acid, in combination with metals such as Co, Cu, Ni, and Zn. Antimicrobial and antifungal properties of metal ions have been known and explored for many years [36]. The antimicrobial activity of the metal ions is linked with the cell membrane destabilization and disruption of the bacterial membrane permeability. Whereas the antifungal effect of the metal ions is mainly due to the disorder of ergosterol biosynthesis. The application of metal ions alone as a preservative agent or drug compound is problematic because they exhibit cytotoxic activity to human cells. The combination of metal ions and CA may be a promising strategy to obtain antimicrobial agent safety for humans. 

In our study, among examined complexes, CA–Zn exhibited the weakest antimicrobial effect, especially against *L. rhamnosus*, *E. coli*, and *P. vulgaris*, when complex CA–Co is characterized by the strongest inhibitory effect against the all of tested microorganism with the exception of *E. coli* and *S. boulardii*. Gałczyńska et al. pointed the fact that Co and Cu-based complexes have an antibacterial and antifungal effect [37]. These properties depend on the mechanism of transport through the bacterial/fungal cell membrane and more precisely, through Ca^2+^ channels in cellular membranes. 

In addition, it should be noted that, the transport of a substance into a bacterial cell depends on the relationship between the individual metals. For example Co may have antagonistic effect against Fe(III). As a result of interaction of Co with a siderophore (pyoverdine) which is produced by *P. aeruginosa*, decrease in ferric ion availability for bacteria cells may be observed. This phenomenon could be connected with the highest CA–Co toxicity observed for *P. aeruginosa*. In our study *C. albicans* appears to be the most sensitive on CA–Co and CA–Ni treatments. Castillo et al. indicate that chemical complexes with Co and Cu have better antimicrobial effect as compared to those containing zinc [38]. In our study, the similar results for CA–Cu were obtained for *C. albicans*, *E. coli*, *P. vulgaris*, and *S. epidermidis*. The probable cytotoxicity effect of metal complexes against bacteria and fungi may be connected with metal complex–membrane components interaction. The study by Lv et al. showed that ergosterol present in *C. albicans* cell membrane together with numerous proteins creates lipid rafts, which are responsible for hyphae morphogenesis, polarization, and membrane fusion during endocytosis [39]. Therefore, treatment of cells with antifungal agents may result in the destruction of lipid rafts in filamentation and thus disrupt the synthesis of ergosterol and hyphae. In turn, antibacterial ability of metal complexes with CA may be connected with changes in membrane properties such as its rigidity and permeability due to membrane protein destruction [33]. 

The previous studies have indicated that the antimicrobial capacity of polyphenolic compounds depends on their properties and structure. As reported by Murcia et al., antibacterial and antifungal responses to treatments may due to different lipophilic properties and dipole moments of examined complexes [40]. Moreover, research conducted by Wu et al. (2013) demonstrated that antimicrobial ability is associated with a negative correlation between the relative hydrophobicity and the numbers of hydroxyl groups in some polyphenolic compounds (flavonoids) [33]. 

Phenolic compounds, such as cichoric acid, consist of a group of compounds of natural plant origin with significant and proved antioxidant activity. They prevent many diseases, including cancer, and cardiovascular and neurodegenerative diseases, mainly by reducing oxidative stress [41,42,43]. Polyphenolic compounds can prevent oxidative stress by inhibiting ROS (Reactive Oxygen Species), but on the other hand they may also act as a prooxidant mainly in the presence of transition metals. Prooxidant activity of polyphenolic compounds is conditioned by many factors, including high concentration of polyphenolic compound, high pH, and the presence of transition metals, such as Cu and Fe [44,45]. Two of the above mentioned factors are especially important—an acidic pH and the number of hydroxyls in aromatic rings. The solubility and stability of reduced forms of transition metals is determined by acidic pH of the solution and the higher number of hydroxyl substitutions, mainly in *ortho* position, determines stronger prooxidant properties [46]. 

Among trace minerals, chromium, cobalt, selenium, iron, manganese, molybdenum, copper, and zinc can be mentioned. Some of them, particularly transition metal ions such as copper, are involved in oxidative reactions in the presence of other compounds, such as polyphenols [47]. One of the most important microelements is copper, which plays an important role in various physiological functions. In cancer cells its concentration is significantly higher than in normal cells, which subsequently makes them less resistant to the prooxidant activity of polyphenols [48,49]. It is in accordance with our results indicating that copper complexed with cichoric acid significantly decreases relative cell viability in every analyzed cell line except for MCF-7 after 24 h incubation. We observed that CA complexed with Cu as compared to free CA caused significantly higher declines in cell viability. It is in agreement with literature data indicating that in the presence of copper prooxidant activity of polyphenols is supposed to progress through the generation of a high level of ROS. It is well known that cancer cells generate oxidative stress, which stimulates their proliferation, but significantly high level of ROS and RNS (Reactive Nitrogen Species) may cause cancer cell damage and death. In the presence of Cu and polyphenols such as CA, cancer cells produce large amount of free radicals which cause DNA damage and apoptosis in cancer cells. The vast majority of studies regarding possible prooxidant activity of polyphenols were conducted in vitro and in the presence of copper as a catalyzer of oxidative reactions [47].

Obtained results indicate that the most biologically active compound was CA complexed with Zn. It was especially cytotoxic against melanoma, colorectal adenocarcinoma, and glioblastoma cell lines. Although, according to the literature data zinc is rather an antioxidant, its overdoses may result in prooxidant activity [50]. Borovansky et al. indicated that melanoma cells are uniquely susceptible to increases of certain divalent metal salts, for example Zn(II). They demonstrated that compounds containing zinc in their structure may induce melanoma cell death at concentrations several times lower than those that are lethal to melanocytes [51]. We observed that zinc complexed with CA was very effective in decreasing relative melanoma cell viability even at lower analyzed concentrations. In fact, the CA–Zn complex was the most efficient from all the analyzed complexes. It could be related with the high susceptibility of melanoma to Zn activity, which was also mentioned by Farmer et al. [52].

Similar results were observed for Ni and Co complexes with CA. Both of them are essential trace elements for the human body, but scarce data is available on the cytotoxic and prooxidant activity of Co and Ni in the presence of natural antioxidants such as polyphenolic compounds. Chen et al. indicated that Ni complexes with polyphenolic ligands exhibited three times stronger responses than a parent compound in the human colon carcinoma cell line (SW620) and the lowest IC_50_ values against the human breast carcinoma cell line (MDA-MB-435) [19]. Our results indicated statistically significant response in MDA-MB-231 breast cancer cell line viability observed as a decrease in analyzed parameter especially after 24 h treatment with CA–Ni complexes. Chen et al. noticed also that anti-proliferative activities of polyphenol–metal complexes were stronger than cisplatin, which was used as a positive control in this experiment. Nickel complexes with CA, similarly as in the case of CA–Zn complexes, were significantly efficient against melanoma, colorectal adenocarcinoma, and glioblastoma cell lines, as compared to three analyzed breast cancer cell lines, which were more resistant to studied compounds. Song et al. suggested that affinity of Ni complexes to DNA may play an important role in determining their anticancer activity [53]. It is possible that complexes synthesized in our laboratory also act through inducing alterations in the DNA structure. However, even though literature data indicate that complexes of polyphenols with selected metals are selective towards cancerous cells over normal cells, we observed different effects. In normal healthy fibroblasts CA–metal complexes exhibited a significant toxicity level as compared to control untreated cells and as compared to free CA. Similarly, as we have shown in our previous work, CA has positive influence on normal healthy cells, stimulating their proliferation and decreasing oxidative stress level, even in the presence of strong prooxidants [22]. 

According to Baile et al. the most effective are copper complexes where pyridine-type ligands (pyridine, bipyridine, phenanthroline, etc.) are present and such where copper(I) ion is coordinated to phosphine ligands. Copper complexes exhibit an excellent antiproliferative effects in cancer cells, which may result from their ability to generate reactive oxygen species [4]. Our results are also in accordance with the other literature data indicating that cichoric acid has a strong growth inhibitory effect against cancer cells resulting from pro-apoptotic effect [54]. We observed that the addition of metals significantly enhanced antiproliferative activity of CA, therefore we conclude that CA–metal complexes have higher efficacy than free polyphenol compound. Literature data showed that daily consumption of *Echinacea*, which is a main source of CA, may be a prophylactic and attenuates leukemia studied in mouse models [55]. In conclusion, the most significant response for CA and CA–metal complexes treatment among three breast cancer cell lines we observed in MCF-7 and ZR-75-1 cell lines. In the MDA-MB-231 cell line we didn’t observe any significant changes as compared to control untreated cells. In general, among all tested cancer cell lines, breast cancer cells were more resistant to studied compounds that the other types of cancer cells. 

Although cichoric acid reveals many beneficial properties and its complexes with selected metals exhibit anticancer and antibacterial properties, it should be mentioned that according to the literature data bioavailability of hydroxycinnamic acids is rather low. Majority of studies regarding hydroxycinnamic acids bioavailability are focused in caffeic acid and chlorogenic acid. The definition of bioaccessibility consists mainly of the estimation of relative amounts of compound which could be released from the food matrix during digestion and could be available for absorption. Literature data indicate that cichoric acid is characterized by rather low bioaccessibility, which is very low in the mouth and stomach steps, but it recovers during the intestinal digestion phase, probably because of the pH changes [56]. 

Complexing drugs with metals is a well-known and common procedure that has been gaining more and more attention in recent years. Complexing some therapeutic compounds, such as polyphenols, can improve their physicochemical and pharmacological properties or reduce their potential side effects. In general, phenolic compounds are often and effectively used to form chelate complexes with various metal ions [57,58].

## 5. Conclusions

In conclusion, the results of the presented study demonstrated, for the first time, that the treatment with newly synthesized CA-metal complexes has anticancer and antimicrobial effects, which was demonstrated in seven different cell lines, five bacterial strains, and pathogenic yeast-like fungi *C. albicans* and yeast *S. boulardii*. The presented study indicates that CA–metal complexes could be potentially effective supplementary tools in anticancer therapy; however they should be used with caution and their activity should be analyzed in other normal healthy cell lines. This is due to their potentially toxic action in fibroblast cells. They have also preservative properties and positive influence on normal non-pathogenic microorganisms, which was demonstrated in selected microbial strains, therefore they may serve as food preservatives of natural origin with cytoprotective properties. There is a huge amount of research and literature data on the anti-tumor effects of polyphenolic compounds. However, although complexing these compounds with selected metals significantly improves their anti-cancer activity, the amount of research conducted on complexes of polyphenols with metals is much smaller. Moreover, none of these complexes have yet entered the phase of clinical trials. This may be due to many problems caused by the presence of a metal ion; among others, an important problem is the stability of such compounds and their solubility in physiological solvents. Therefore, further studies are necessary to determine the mechanisms by which the analyzed compounds affect a reduction in viability of tumor cells and in normal cells, and cells of bacterial and fungal origins. 

## Figures and Tables

**Table 1:** Elemental analysis for metal complexes with cichoric acid.

Empirical Formula	Yield	%H (Theoret)	%C (Theoret)	%H (Exp)	%C (Exp)
[C_22_O_12_H_16_Zn_2_] ·4H_2_O	70–75%	3.60	39.59	3.69	39.80
[C_22_O_12_H_16_Ni_2_] ·4H_2_O	70–80%	3.67	40.41	3.56	39.76
[C_22_O_12_H_16_Cu_2_] ·4H_2_O	70%	3.62	39.81	3.57	39.13
[C_22_O_12_H_16_Co_2_] ·4H_2_O	70–75%	3.67	40.38	3.59	40.05

**Table 2:** Wavenumbers (cm^−1^), intensities, and assignments of bands occurring in the IR (Infrared) (KBr and HF –Hartrre-Fock method) and Raman spectra of cichoric acid, sodium salt, and 3D metal complexes.

Cichoric Acid				Sodium Salt				Complexes						Assignments
								Copper			Nickel	Zinc	Cobalt	
Experimental		Theoretical		Experimental		Theoretical		Exp	Theoret		Exp	Exp	Exp	
IR	Raman	HF	Int	IR	Raman	HF	Int	IR	HF	Int	IR	IR	IR	
1746 m		1809	331.95											ν_as_COOH_tart_
1716 s		1797	512.19											ν_as_COOH_tart_
1682 vs	1681 s	1744	18.96	1699 s	1703 w	1735	7.47	1721 s	1736	9.23	1698 s	1692 m		νC = O_caff_. νC = C_alcaff_
1624 s	1627 vs	1742	697.97			1732	686.64		1731	669.21				νC = O_caff_. νC = C_alcaff_
				1626 vs		1580	1255.35	1626 s	1570	1105.71	1630 s	1629 vs	1605 s	ν_as_COO^-^
				1385 s	1381 vw	1447	204.62	1384 m	1446	185.54	1385 m	1384 s	1385 m	ν_s_COO^-^
				853 w	860 vw	870	18.44	868 w	871	10.97	851 w	853 w	851 m	β_s_COO^-^
						869	8.1		869	20.35				β_s_COO^-^
698 vw	699 vw	739	2.12			746	3.41		745	4.05	698 w	695 m		γCOOH
680 w	685 vw	727	4.36	688 w		727	1.11		726	2.16	691 m		685 m	def_ring_. γCOOH
				521 w		539	40.51	521 m	536	39.39	520 m	520 m	521 m	β_as_COO^-^
						472	39.33		471	46.44				β_as_COO^-^
504 w	504 vw	517	7.63											γO-H_caff_

ν-stretching vibrations, β-bending in-plane, γ-bending out-of-plane, def_ring_-deformation of the ring in-plane, s-symmetric oscillations, as-asymmetric oscillations, caff-caffeic acid, tart-tartaric acid.
